# Distinct insulin sensitivity profiles revealed by clustering based on continuous glucose monitoring data in people living with type 1 diabetes

**DOI:** 10.3389/fendo.2026.1963929

**Published:** 2026-09-28

**Authors:** Svjatoslavs Kistkins, Anastasija Luganceva, Sergejs Lobanovs, Timurs Mihailovs, Aleksejs Fedulovs, Leonora Pahirko, Valdis Pīrāgs, Othmar Moser, Harald Sourij, Jelizaveta Sokolovska, Dmitrijs Bliznuks

**Affiliations:** 1 Research Institute of Pauls Stradins Clinical University Hospital, Riga, Latvia; 2 Faculty of Medicine and Life Sciences, University of Latvia, Riga, Latvia; 3 Institute of Applied Computer Systems, Riga Technical University, Riga, Latvia; 4 Institute of Human Movement Science, Sport & Health, Exercise Physiology, Training & Training Therapy Research Group, University of Graz, Graz, Austria; 5 Interdisciplinary Metabolic Medicine Trials Unit, Division of Endocrinology and Diabetology, Medical University of Graz, Graz, Austria

**Keywords:** cluster analysis, continuous glucose monitoring, diabetes mellitus, type 1, insulin resistance, personalized medicine

## Abstract

**Background:**

Continuous glucose monitoring (CGM) has improved glycemic management in Type 1 Diabetes Mellitus (T1DM). However, interpretation of CGM data remains challenging. CGM data clustering may support individualized management and identify insulin resistance.

**Aim:**

This study aimed to cluster CGM data based on interstitial glucose levels (2.2–27.8 mmol/L) and glucose variability, and to compare the identified clusters with biochemical and anthropometric markers of insulin sensitivity.

**Methods:**

CGM data from 75 T1DM patients and 20 subjects without diabetes were analyzed using Uniform Manifold Approximation and Projection (UMAP) for dimension reduction, followed by K-means clustering. Estimated glucose disposal rate (eGDR) was calculated to assess insulin sensitivity.

**Results:**

Seven clinically distinct CGM phenotypes were identified: Normoglycemia-in-Diabetes (N), Normoglycemia-in-Non-Diabetes (No), Mild Hyperglycemia (MiH), Moderate Hyperglycemia (ModH), Severe Hyperglycemia (SeH), Hypoglycemia-Associated Normoglycemia (HAN), and Hypoglycemia-Associated Hyperglycemia (HAH). The *SeH* cluster demonstrated the highest median average glucose level (13.83 [13.07;14.12] mmol/L), while *HAH* exhibited the highest median coefficient of variation (50 [44;52]%). The lowest median average glucose level was observed in *HAN* (6.14 [5.95;6.17] mmol/L). A marginally significant difference in ALT levels was observed between SeH and No (p=0.07). eGDR differed significantly between *SeH*/*HAN* (p=0.001), *ModH*/*HAN* (p=0.019), and *MiH*/*HAN* (p=0.043), with marginally significant differences between *HAN*/*HAH* (p=0.063). Triglycerides differed significantly between *HAN/SeH* (p=0.029), *HAN*/*MiH* (p=0.024).

**Conclusion:**

This exploratory CGM-based clustering approach identified phenotypically distinct T1DM subgroups with differing insulin sensitivity profiles. These preliminary findings require validation in larger independent cohorts. If confirmed, such phenotyping may support individualized diabetes management and identification of insulin resistance.

## 1 Background

Continuous glucose monitors (CGMs) revolutionized type 1 diabetes mellitus (T1DM) management, shifting from traditional metrics like HbA1c to precise indicators such as time in range (TIR, 3.9–10.0 mmol/L) (1). Despite technological advancements, a substantial proportion of individuals with T1DM still fail to achieve recommended glycemic targets (2). Improved insulin treatments, sedentary lifestyles, and unhealthy diets have increased insulin resistance and metabolic syndrome prevalence in T1DM, heightening risks of retinopathy, nephropathy, and cardiovascular disease (3).

By 2021, 8.4 million people worldwide lived with T1DM, a figure projected to reach 17.4 million by 2040. Many struggle to meet individualized treatment goals, emphasizing the need for improved management strategies (4–6). Recent studies (7, 8) highlight the role of machine learning in refining patient stratification based on CGM data.

Kovatchev et al. (7) published an article proposing a new model of patient glycemic control through the introduction of a finite and fixed set of clinically similar clusters. Kovatchev’s team identified 32 clusters in healthy, type 1 and 2 diabetes patients on different treatments, proposing a new research avenue identifying clusters with clinical markers in these populations (7). Related work by Kovatchev and colleagues includes the development of the UVA/PADOVA Type 1 Diabetes Simulator, which has also been used by our group to train and evaluate machine-learning models for interstitial glucose-level classification (9, 10). Additionally, recent studies have compared TIR (3.9–10.0 mmol/L) with TITR.

(time in tight range) - defined as 3.9–7.8 mmol/L, highlighting a new and more stringent criterion for glycemic control (11, 12). In summary, new approaches are needed to enhance clinically relevant decision-making in the management of subjects with T1DM. One of these approaches could be cluster-related treatment strategies and prediction of cluster-related diabetes outcomes.

## 2 Materials and methods

### 2.1 Patient cohort and data preparation

CGM data were acquired from the longitudinal LatDiane study, which forms part of the InterDiane Consortium. A more detailed description of the InterDiane Consortium and the LatDiane cohort has been published previously (13). For this secondary analysis of the LatDiane project, we analyzed data from 75 patients with T1DM and 20 individuals without diabetes who constituted the control group.

The study was approved by the local ethics committee (approval Nos. 01-29.1/3, A-17/19-10–17 and 01-29.1.2/927). The study adheres to the principles outlined in the 1964 Declaration of Helsinki and its subsequent amendments. Written informed consent was obtained from all participants before inclusion in the study. For participants with T1DM, inclusion criteria were T1DM diagnosed before the age of 40 years, insulin treatment initiated within one year of diagnosis and C-peptide levels below 0.3 nmol/L (defined within InterDiane consortium), T1DM duration at least 8 years (13). The control group consisted of otherwise healthy individuals with normal glucose metabolism according to the American Diabetes Association criteria, confirmed by fasting glucose and HbA1c levels (14). The control group consisted of spouses and friends of the recruited participants with T1DM, as well as university personnel and students. No formal matching procedure based on age, sex, or BMI was applied. The exclusion criteria for both groups were pregnancy, inflammatory bowel disease (Crohn’s disease or ulcerative colitis), celiac disease (detected through serum transglutaminase IgA screening), recent acute intestinal infection (within 2 months), clinical signs of acute inflammation, and fever, history of chronic kidney disease apart from diabetic kidney disease (DKD). For patients with diabetes, additional exclusion criterion was diabetes duration<8 years. On the study day, participants underwent anthropometric assessment, CGM sensor placement, and blood and urine sampling for clinical biochemistry. Both T1DM patients and control group followed their usual diet. All clinical chemistry measurements from blood samples were done in a certified clinical laboratory.

### 2.2 CGM

FreeStyle Libre Pro iQ Sensors (Abbott, Wiesbaden, Germany) were used for 14 days. CGM data were interpreted using established metrics: Time in Range (TIR; tissue glucose 3.9-10.0 mmol/L), Time Above Range (TAR; >10.0 mmol/L), and Time Below Range (TBR;<3.9 mmol/L). Coefficient of variation (CV) values ≥ 36% were categorized as high glucose variability (GV) (1).

### 2.3 Clusterization of CGM raw data

The CGM data included 123,319 measurements, with a mean of 9.18 ± 4.80 mmol/L, and a median of 7.8 mmol/L with an interquartile range from 5.6 mmol/L (Q1) to 11.8 mmol/L (Q3).

We employed data-driven cluster analysis on raw CGM data, utilizing an unsupervised machine learning algorithm (UMAP) for dimensionality reduction based on the original dataset containing glucose value distributions. UMAP identifies natural groupings in the data, uncovering hidden patterns without prior labels and enabling the identification of distinct patient profiles based on CGM data. UMAP calculates distances between data points in multidimensional space and projects it to a two or three dimensional space that could be evaluated visually (**Supplementary Figure S1**). CGM glucose values were categorized into six TIR levels, two TBR levels and three TAR levels, using 1 mmol/L intervals. These glucose distribution features were subsequently used as input for UMAP. More specifically, UMAP was used to project the high-dimensional glucose distribution space into a 2D representation, thereby preserving local and global data structure while enabling visual inspection of natural data groupings.

The number of clusters of patients with T1DM was evaluated using both silhouette score analysis and the elbow method applied to K-means clustering. Silhouette analysis indicated an optimum of K = 11; however, the elbow method (**Supplementary Figure S2**), based on the distortion metric (within-cluster sum of squares), showed a clear elbow at K = 6, indicating that further increases in the number of clusters provided comparatively limited improvement in within-cluster compactness. Importantly, the six-cluster solution also provided a more clinically interpretable representation of the observed glycemic patterns. Therefore, K = 6 was selected as the final number of clusters based on the combination of the elbow criterion and clinical interpretability, rather than by *post hoc* merging of the 11 clusters identified by silhouette analysis.

To assess the robustness of the dimensionality-reduction step, UMAP was initialized using multiple random seeds, yielding consistent embeddings across runs (umap-learn Python library, version 0.3.10). Once consistency was confirmed, the random seed was fixed to ensure reproducibility. Each patient was characterized based on average interstitial glucose level, standard deviation (SD), and coefficient of variation (CV), while clusters were characterized by the median values of these parameters (**Table 1**). The resulting six clusters represent distinct and clinically interpretable glycemic patterns related to glucose level and variability in patients with diabetes. In total, six clinically relevant exploratory clusters of patients with diabetes were identified, in addition to a group of healthy participants without diabetes (**Table 1**, **Figure 1**).

**Table 1:** Median of average, SD and CV of glucose values over all patients of CGM clusters.

CGM-derived phenotype (n)	Abbreviation	Average glucose, mmol/L, median (Q25; Q75)	SD, mmol/L, median (Q25; Q75)	CV, %, median (Q25; Q75)
Normoglycemia-in-Diabetes (11)	N	7.42	2.83	38
		(7.15; 7.55)	(2.50; 2.98)	(34; 39)
Normoglycemia-in-Non-Diabetes (20)	No	5.7	0.91	16
		(5.49; 6.02)	(0.76; 0.98)	(14; 19)
Mild Hyperglycemia (15)	MiH	8.91	3.31	37
		(8.65; 9.40)	(3.11; 3.58)	(36; 40)
Moderate Hyperglycemia (14)	ModH	10.67	4.25	37
		(10.49; 11.33)	(4.01; 4.49)	(36; 40)
Severe Hyperglycemia (17)	SeH	13.83	5.35	37
		(13.07; 14.12)	(4.75; 5.72)	(35; 42)
Hypoglycemia-Associated Normoglycemia (5)	HAN	6.14	2.55	41
		(5.95; 6.17)	(2.39; 2.69)	(39; 45)
Hypoglycemia-Associated Hyperglycemia (13)	HAH	8.87	4.18	50
		(8.25; 9.52)	(3.95; 4.97)	(44; 52)

After clusterization and cluster grouping, 6 clusters of subjects with diabetes and one cluster of control subjects were obtained.

**Figure 1:**
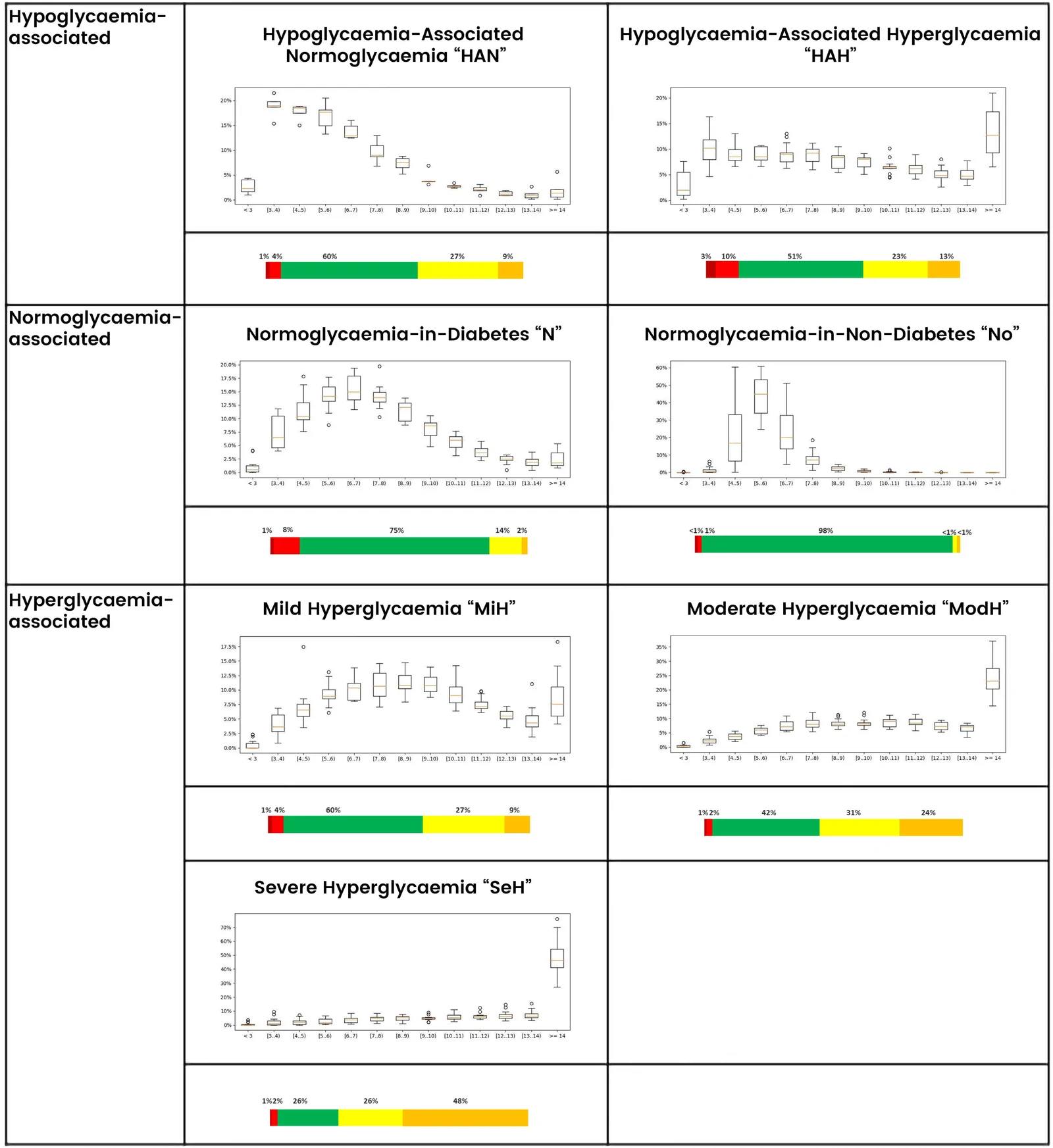
Distribution of time spent in glucose ranges. The x-axis shows glucose levels in intervals from<3 mmol/L to >14 mmol/L, while the y-axis represents the percentage of time individuals in the normoglycemic cluster spent in each range. Each boxplot depicts the distribution of time. This figure also breaks down the percentage of time spent in different glucose categories: Severe hypoglycemia (<3.0 mmol/L, red); mild hypoglycemia (3.0–3.9 mmol/L, light red). Target range (3.9–10.0 mmol/L, green). Mild hyperglycemia (10.0–13.9 mmol/L, yellow); severe hyperglycemia (>13.9 mmol/L, orange).

### 2.4 Statistical analysis

Statistical analysis was performed using a significance level of p ≤ 0.05. Additionally, p-values ranging from 0.05 to 0.1 were interpreted as indicating a marginally significant difference. Normal distribution of study variables was assessed using the one-sample Kolmogorov-Smirnov test and data distributions were visually represented using histograms. Non-parametric statistical tests were used to compare clusters based on CGM data, specifically, the Mann-Whitney U test, the Kruskal-Wallis H test and *post hoc* Dunn’s test were employed for different anthropometric and biochemical data involving two or more groups. When multiple pairwise comparisons were performed, Bonferroni correction was applied to adjust p-values and control for type I error. In addition, supplementary unadjusted pairwise comparisons were conducted for exploratory purposes to identify potential patterns and generate hypotheses in this relatively small sample. Results of these exploratory analysis are reported separately and should be interpreted with caution due to the increased risk of type I error. The inclusion of liver enzymes in these exploratory analysis was motivated by emerging evidence linking CGM-derived glycemic metrics and insulin sensitivity with metabolic dysfunction-associated steatotic liver disease (MASLD) in people with T1DM (15, 16). We hypothesized that each cluster exhibits distinct anthropometric, biochemical, or clinical characteristics: hypoglycemia events, liver enzymes (ALT), HbA1c, eGDR, triglycerides, HDL, basal insulin per kg body weight (**Supplementary Table S1**). A hypoglycemic event was defined as sensor glucose<3.9 mmol/L for at least 15 consecutive minutes. For each participant, the number of hypoglycemic events and the duration of each event were calculated over the CGM recording period. The mean event duration was then calculated for each participant, and these participant-level mean values were compared across clusters. Participants with no hypoglycemic events were assigned a value of 0 minutes. Time below range was additionally classified as level 1 hypoglycemia (3.0–3.8 mmol/L) and level 2 hypoglycemia (<3.0 mmol/L) (**Figure 2**, **Supplementary Table S2**). The eGDR was calculated using the following equation, which incorporates hypertension diagnosis (hypertension was coded as 0 or 1 based on blood pressure ≥140/90 mmHg), HbA1c levels, and waist-to-hip ratio:

**Figure 2:**
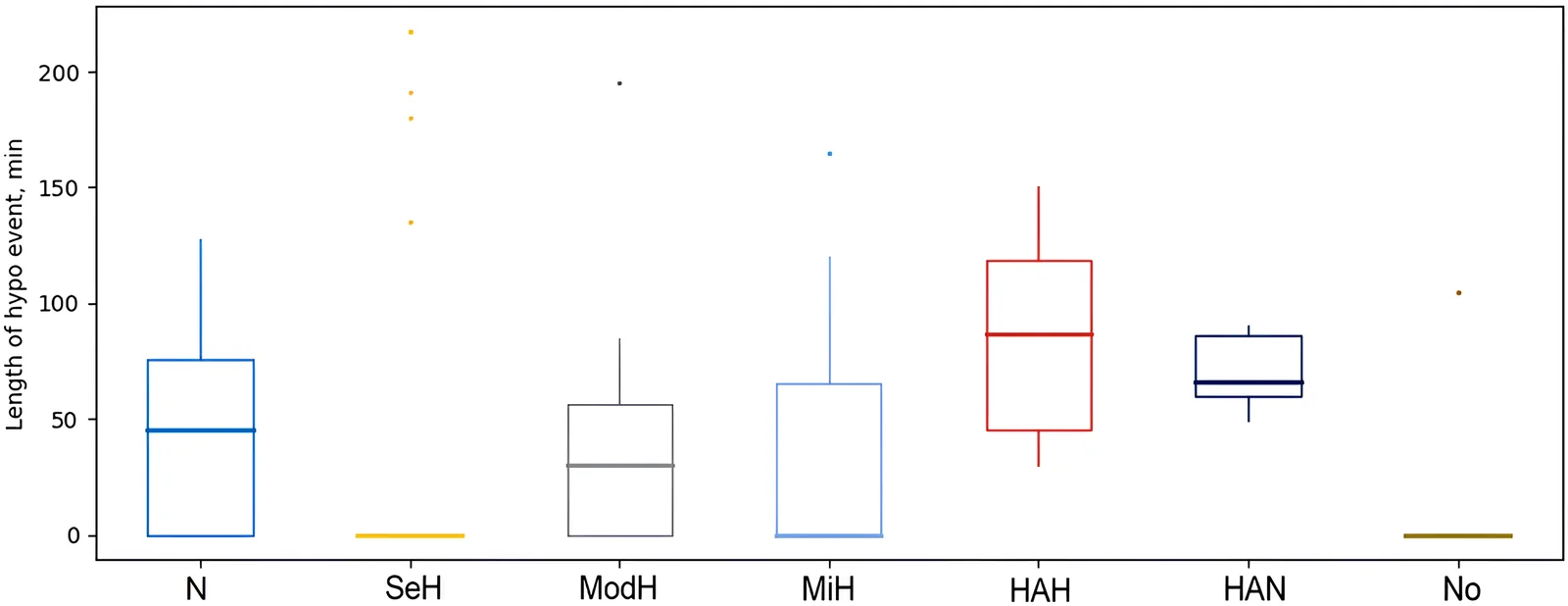
Hypoglycemia event duration in minutes. The graph presents hypoglycemic event durations per cluster, calculated as median of individual patient averages.

eGDR=24.4−12.97×Waist/Hips−3.39×Hypertension−0.6×HbA1c(%) (17).

Effect sizes (η2) with 95% confidence intervals (CI) were calculated for the Kruskal-Wallis H test to quantify the differences between groups.

## 3 Results

### 3.1 Characteristics of study participants

The study included 75 (44, 58.7% women) patients with T1DM. The median age was 44 (34; 53) years, median BMI was 24.5 (22.95; 28.3) kg/m². 41 patients (54.7%) were non-smokers. Hypertension was present in 44 participants (58.7%), and 14 (18.7%) had cardiovascular disease (CVD). The median duration of diabetes was 25 (16.5; 33) years, and the median HbA1c was 8.2 (7.3; 9.55) %. 39 participants (52.0%) had retinopathy, and 22 (29.3%) had autoimmune thyroid disease. Macroalbuminuria (albumin levels >30 mg/mmol (18)) was observed in 12 individuals (16%). The control group comprised 20 participants without diabetes, of whom 14 (70.0%) were women. Their median age was 37.0 (34.25; 41.0) years, and their median BMI was 23.65 (21.53; 24.95) kg/m². Fifteen participants (75.0%) were non-smokers. Hypertension was present in 3 participants (15.0%), autoimmune thyroid disease in 2 (10.0%), and another autoimmune disease in 1 (5.0%). None of the control participants had CVD or macroalbuminuria. Their median HbA1c was 5.15% (4.88; 5.33), corresponding to 33 (30; 35) mmol/mol.

### 3.2 Characteristics of CGM-derived phenotypes

After clusterization and cluster grouping, 6 CGM-derived phenotypes of subjects with diabetes and one phenotype of control subjects were obtained (**Table 1**, **Figure 1**).

Normoglycemia-associated CGM-derived phenotypes: Normoglycemia-in-Diabetes (“N”) and Normoglycemia-in-Non-Diabetes (“No”) represent distinct CGM-derived phenotypes with clear and consistent patterns in their glycemic levels, showing minimal overlap or ambiguity in classification. True normoglycemic CGM-derived phenotype or Cluster “N” represents individuals who consistently maintain normal glucose levels without significant deviations. Patients of the cluster “N” spent more than 75% of the time below 10 mmol/L (median of average glucose levels is 7.42 mmol/L). The Normoglycemia-in-Non-Diabetes CGM-derived phenotype serves as a” Control” and baseline for comparison, representing individuals with well-regulated and stable blood glucose levels, often considered the healthy reference group (median of average glucose levels - 5.70 mmol/L).

Hyperglycemia-associated CGM-derived phenotypes: Mild Hyperglycemia (“MiH”), Moderate Hyperglycemia (“ModH”) and Severe Hyperglycemia (“SeH”) represent exploratory clusters of patients whose glycemic profiles include a meaningful hyperglycemic component. The Mild Hyperglycemia cluster (“MiH”) characterizes individuals who predominantly maintain near-normal glucose levels but exhibit recurrent excursions into the hyperglycemic range, with a median average glucose of 8.91 mmol/L. The Moderate Hyperglycemia CGM-derived phenotype (“ModH”) describes individuals with predominantly elevated glucose levels and a tendency toward normoglycemia, reflected by a median average glucose of 10.67 mmol/L—lower than the Severe Hyperglycemia cluster but substantially above the normoglycemic range. “SeH” CGM-derived phenotype includes those with consistently high glucose levels, indicative of hyperglycemia. The true hyperglycemic CGM-derived phenotype “SeH” had the highest mean glucose level (median of average glucose levels - 13.83 mmol/L) with 75% of the time above 10 mmol/L.

Hypoglycemia-associated CGM-derived phenotypes: Hypoglycemia-Associated Hyperglycemia (“HAH”) and Hypoglycemia-Associated Normoglycemia (“HAN”) represent exploratory clusters of patients n whom hypoglycemic episodes constitute a clinically significant component of the overall glycemic burden. The Hypoglycemia-Associated Hyperglycemia CGM-derived phenotype (“HAH”) is characterized by marked glucose variability with alternating hypoglycemic and hyperglycemic excursions, reflected by the highest median CV among all clusters (50 [44;52]%) and a median average glucose of 8.87 mmol/L. The Hypoglycemia-Associated Normoglycemia CGM-derived phenotype (“HAN”) comprises individuals who maintain near-normal average glucose levels but experience recurrent hypoglycemic episodes; this cluster had the lowest median average glucose among all T1DM clusters (6.14 [5.95;6.17] mmol/L).

#### 3.2.1 Hypoglycemia events per cluster

Hypoglycemic event durations differed significantly across the six clusters of patients with diabetes (Kruskal–Wallis, p = 0.0141; **Figure 2**). The longest median hypoglycemic event duration was observed in the Hypoglycemia-Associated Hyperglycemia (“HAH”) CGM-derived phenotype, at 87 (45.00; 118.33) minutes, while more than 75% of patients in the Severe Hyperglycemia (“SeH”) CGM-derived phenotype experienced no hypoglycemic events. *Post-hoc* Dunn’s test confirmed a statistically significant difference in hypoglycemic durations between the SeH and HAH CGM-derived phenotype (p = 0.0255). The comparison between the Mild Hyperglycemia (“MiH”) and HAH CGM-derived phenotypes showed a marginally significant difference (p = 0.0704), which should be interpreted cautiously given the small cluster sizes and absence of multiple comparison correction. The second-longest median duration was observed in the Hypoglycemia-Associated Normoglycemia (“HAN”) CGM-derived phenotype (66 minutes [60.00; 85.90]). Neither the HAH nor HAN exploratory clusters included patients free of hypoglycemic events. The Normoglycemia-in-Diabetes (“N”) and Moderate Hyperglycemia (“ModH”) CGM-derived phenotype showed moderate event durations (45 [0.00; 75.75] and 30 [0.00; 56.25] minutes, respectively). These findings highlight clinically meaningful differences in hypoglycemia burden across phenotypic clusters and may support the development of more tailored glycemic management strategies.

### 3.3 Characteristics of clusters based on anthropometric and biochemical data

**Supplementary Table S1**; **Figure 3** present clinical characteristics of patients in each of the clusters.

**Figure 3:**
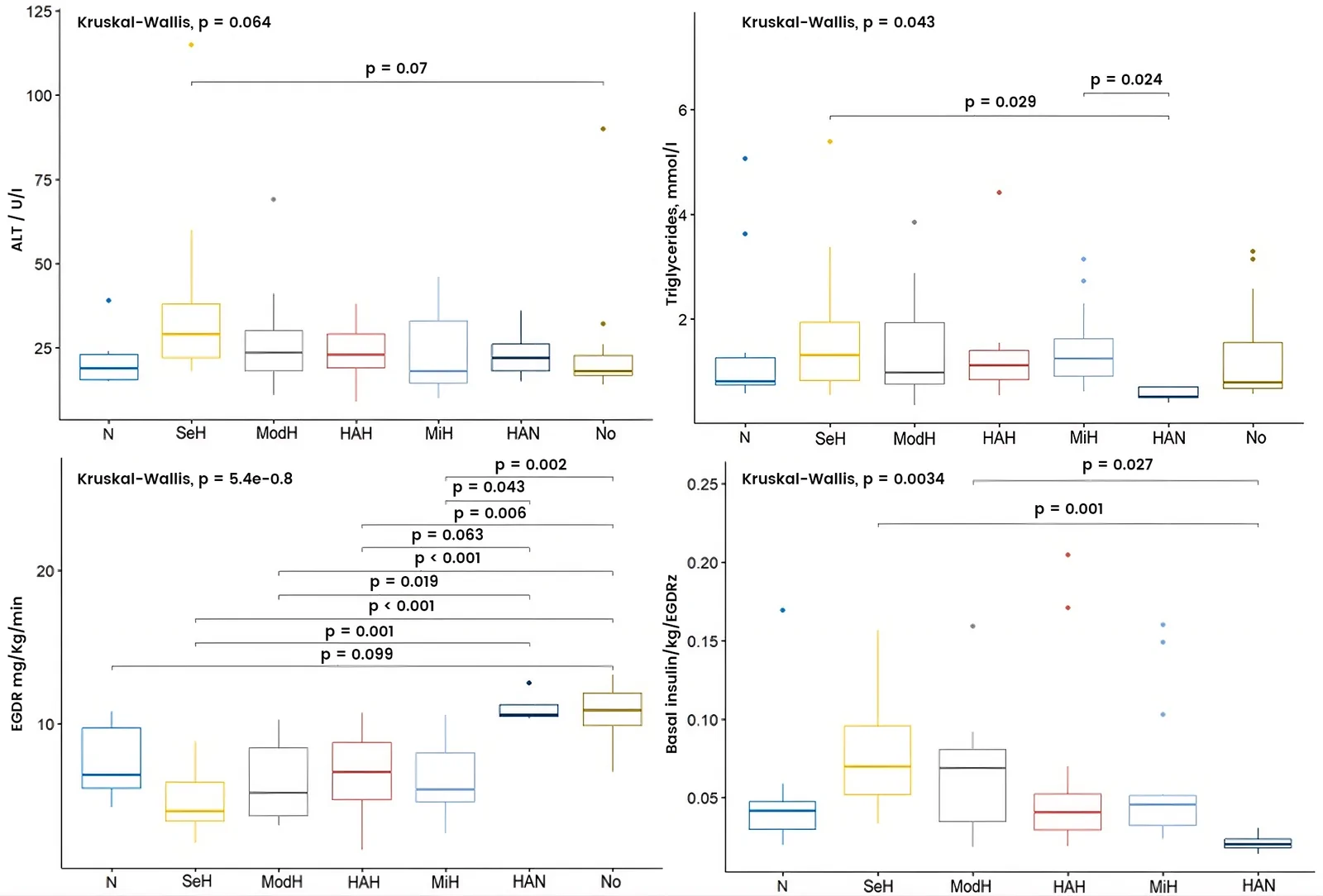
ALT, TG, eGDR, basal insulin/kg/eGDR according to each separate cluster. Bonferroni correction was applied to adjust p-values and control for type I error.

#### 3.3.1 Liver enzymes (ALT)

ALT levels showed marginally significant differences across the seven clusters (Kruskal–Wallis, p = 0.064), with a moderate effect size accounting for 6.7% (95% CI: 1%, 28%) of the variance. *Post-hoc* Dunn’s test identified marginally significant difference between the Severe Hyperglycemia (“SeH”) and the “No” CGM-derived phenotype (p = 0.07); this finding should be interpreted with caution as it did not reach the pre-specified significance threshold. No significant differences in ALT were detected in the analysis restricted to T1DM patients (p = 0.12). To complement formal *post-hoc* testing and explore patterns in this small-sample dataset, unadjusted pairwise comparisons were also performed. These analyses identified additional nominally significant differences—between the Severe Hyperglycemia (“SeH”) and Normoglycemia-in-Diabetes (“N”) CGM-derived phenotypes (unadjusted p = 0.011), and between “SeH” and the Mild Hyperglycemia (“MiH”) CGM-derived phenotypes (unadjusted p = 0.012)—though these must be interpreted with caution given the elevated risk of Type I error.

#### 3.3.2 Triglycerides

Triglyceride levels differed significantly across exploratory clusters (Kruskal–Wallis, p = 0.043), with a moderate effect size (η² = 0.079; 95% CI: 0.01, 0.29). *Post-hoc* Dunn’s test identified significant differences between the Severe Hyperglycemia (“SeH”) and Hypoglycemia-Associated Normoglycemia (“HAN”) CGM-derived phenotypes (p = 0.029), and between the Mild Hyperglycemia (“MiH”) and HAN CGM-derived phenotypes (p = 0.024). In supplementary unadjusted pairwise comparisons, the HAN CGM-derived phenotype consistently demonstrated lower triglyceride levels relative to all other exploratory clusters (unadjusted p-values: “N”/”HAN”: p = 0.021; “SeH”/”HAN”: p = 0.001; “ModH”/”HAN”: p = 0.009; “HAH”/”HAN”: p = 0.010; “MiH”/”HAN”: p = 0.001; “No”/”HAN”: p = 0.024). These unadjusted comparisons are exploratory and should be interpreted with caution.

#### 3.3.3 Estimated glucose disposal rate

The Kruskal–Wallis test demonstrated highly significant differences in eGDR across exploratory clusters (H(6) = 44.7, p< 0.001), with a large effect size explaining 44.0% (95% CI: 32%, 61%) of the variance in eGDR.

eGDR levels were lower in most T1DM clusters compared with the non-diabetic control group. Significant differences were observed for the SeH, ModH, HAH and MiH CGM-derived phenotypes, whereas the “N” CGM-derived phenotype showed marginally significant lower eGDR (p = 0.099), and the “HAN” CGM-derived phenotype did not differ significantly from the control group (“No” vs. “SeH”: p< 0.001; “No” vs. “ModH”: p< 0.001; “No” vs. “HAH”: p = 0.006; “No” vs. “MiH”: p = 0.002). This comparison was performed to benchmark the T1DM clusters against a metabolically normal reference group and to contextualize the magnitude of the observed differences in eGDR. However, eGDR incorporates HbA1c, waist-to-hip ratio, and hypertension status and was originally derived and validated in T1DM populations; therefore, this comparison should not be interpreted as demonstrating equivalent differences in directly measured insulin sensitivity between T1DM and non-diabetic individuals.

Within T1DM exploratory glycemic clusters, the “HAN” exhibited significantly higher eGDR values compared to the Severe Hyperglycemia (“SeH”; p = 0.001), Moderate Hyperglycemia (“ModH”; p = 0.019), and Mild Hyperglycemia (“MiH”; p = 0.043) CGM-derived phenotype. A marginally significant difference in eGDR was observed between the HAN and Hypoglycemia-Associated Hyperglycemia (“HAH”) CGM-derived phenotypes (p = 0.063); however, this finding should be interpreted cautiously given the small cluster sizes. These findings suggest that phenotypic clusters defined by hyperglycemia are associated with greater insulin resistance as estimated by eGDR, while the HAN CGM-derived phenotype may represent a metabolically more favorable profile within the T1DM spectrum. However, it is important to recognize that eGDR represents a surrogate, rather than a direct, measure of insulin sensitivity. As its calculation incorporates HbA1c, hypertension status, and waist-to-hip ratio, these constituent variables may systematically differ across CGM-defined glycemic phenotypes. Consequently, the observed association between cluster membership and eGDR may be partially attributable to structural differences in the variables incorporated into the eGDR equation, rather than reflecting an independent association with underlying insulin resistance.

#### 3.3.4 Basal insulin per kg and eGDR

No statistically significant differences in weight-adjusted basal insulin dosage (insulin/kg) were observed across the six T1DM exploratory glycemic clusters, suggesting that insulin requirements based on body weight alone did not differ systematically between glycemic phenotypes.

However, significant differences were identified when basal insulin dosage was adjusted for eGDR (basal insulin per kg/eGDR), reflecting variation in insulin requirements relative to metabolic efficiency. The Kruskal–Wallis test confirmed a significant overall effect (H(5) = 17.6, p = 0.003). *Post-hoc* Dunn’s test showed that the Severe Hyperglycemia (“SeH”) and Hypoglycemia-Associated Normoglycemia (“HAN”) CGM-derived phenotypes differed significantly (p = 0.001), as did the Moderate Hyperglycemia (“ModH”) and HAN CGM-derived phenotypes (p = 0.027). These findings indicate the potential importance of considering metabolic efficiency (as estimated by eGDR) when interpreting insulin requirements across glycemic phenotypes.

## 4 Discussion

Our study applied data-driven cluster analysis to categorize interstitial CGM data and variability, identifying six CGM-derived phenotypes of T1DMpatients and a non-diabetic control group. These findings highlight the importance of considering glycemic patterns and insulin sensitivity markers in T1DM management. The relationship between liver enzymes and glycemic control in T1DM has been well-documented, with elevated liver enzymes associated with poorer glycemic control and increased cardiovascular risk (19–21). eGDR is a valuable tool for assessing insulin resistance in T1DM patients, aiding in the early detection and prevention of diabetic complications (22). Lower eGDR values have been associated with higher insulin resistance levels (23). Our research findings also highlight the importance of lipid levels and their ratios in understanding insulin sensitivity in T1DM patients (24). Low triglyceride levels and high eGDR values were observed in the Hypoglycemia-Associated Normoglycemia (“HAN”) CGM-derived phenotype, suggesting a more favorable metabolic profile compared with other T1DM phenotypes.

Previous studies have shown that CGM can lead to significant improvements in TIR and reductions in average glucose values and estimated glycosylated hemoglobin (25, 26). The incorporation of TITR as a more stringent glycemic target is increasingly recognized as clinically relevant, particularly for populations striving for near-normoglycemia (11, 12). At the same time there is greater heterogeneity in diabetes mellitus subtypes than the classical division, which is solely based on glycemic levels and HbA1c (27). CGM is on the brink of transforming the management of diabetes and improving patient involvement for individuals impacted by this condition (28). The concept of glycemic phenotyping through CGM data is in an active process of clinical validation: Hall et al. introduced the concept of “glucotypes” to describe distinct patterns of glucose dysregulation in non-diabetic individuals (29), while Tao et al. applied multilevel clustering to CGM data in type 2 diabetes (30). Kovatchev and Lobo formalized the concept of Clinically Similar Clusters (CSCs) derived from daily CGM profiles, proposing a finite taxonomy of glycemic patterns applicable across diabetes types and treatment modalities (7). The present study extends this approach to a T1DM cohort incorporating insulin sensitivity estimation via eGDR, which represents an additional analytical dimension not previously integrated in CGM-based clustering frameworks. Furthermore, the association of hyperglycemia-associated clusters with elevated liver enzyme levels is consistent with prior findings regarding the relationship between chronic hyperglycemia and hepatic metabolic dysfunction in T1DM (15, 16, 19–21). The greater heterogeneity in diabetes subtypes beyond classical HbA1c-based classification has been highlighted in recent systematic reviews (27), underscoring the need for more nuanced phenotypic approaches. CGM-based phenotyping may contribute to such refinement, enabling identification of patient subgroups with distinct insulin sensitivity profiles that may benefit from targeted management strategies (27, 28). Transitions between clusters over time could serve as markers of treatment response in future longitudinal studies, though this remains speculative in the absence of longitudinal data. Phenotypes characterized by low eGDR and elevated glucose variability, such as the Hypoglycemia-Associated Hyperglycemia (“HAH”) CGM-derived phenotypes, may warrant closer clinical attention and could represent an interesting target for future intervention studies, though this hypothesis requires prospective evaluation. The physiological determinants of glycemic variability in T1DM are multifactorial and not fully captured by CGM metrics alone; future work incorporating dietary, physical activity, and autonomic nervous system data may further refine phenotypic classification.

The previously mentioned method of identification of Clinically Similar Clusters (CSCs) further advances the widely accepted Time in Range system of metrics, proposed by the International Consensus, by introducing a finite and fixed set of CSCs (7). In our study, all identified clusters showed consistent trends in CGM metrics such as TAR, TIR, TBR. Research has demonstrated that these CGM metrics, specifically %TAR and %TIR, exhibit stronger correlations with Mean Glucose compared to HbA1c, making them sensitive indicators of treatment efficacy (31). In the study we used dimensionality reduction to understand large, high-dimensional datasets of CGM metrics, which is a powerful tool for machine learning practitioners (32).

An exploratory composite measure, calculated as basal insulin dose per kg body weight divided by eGDR (basal insulin/kg/eGDR), was additionally examined to describe weight-adjusted basal insulin requirements relative to estimated metabolic efficiency. This ratio is not an established or validated clinical index, and no reference values have been established. It was therefore analyzed solely as an exploratory measure for hypothesis generation and requires independent validation before clinical interpretation.

Several limitations of the present study must be explicitly acknowledged. First, the small sample size (N = 75 T1DM patients and N = 20 healthy individuals) is the primary limitation of this work, raising concerns about the stability and generalizability of the identified clusters. Recent work across medical applications has highlighted that models developed using single-center, retrospective, or otherwise limited cohorts may show reduced performance when applied to different populations, institutions, devices, or clinical settings, underscoring the importance of independent external validation before clinical translation, including for AI-based approaches in other medical fields (33, 34). This consideration is particularly relevant to studies based on CGM data, where relatively small clinical cohorts are not uncommon. For example, previous research evaluating predictive models for CGM-based classification of hypoglycemia, euglycemia, and hyperglycemia used longitudinal CGM data from only 11 individuals with type 1 diabetes and demonstrated that meaningful predictive modeling and comparison of ARIMA, logistic regression, and LSTM approaches could nevertheless be performed (10). Such studies demonstrate the methodological value of intensively sampled longitudinal CGM data even in small cohorts, while also highlighting the need for larger independent populations when conclusions are intended to be generalized or translated into clinical practice.

Interpretability is also particularly relevant for CGM-based phenotyping, as clinicians need to understand which glucose characteristics and associated clinical variables contribute to cluster assignment before such information could meaningfully support clinical decision-making. In addition, implementation would require evaluation of how cluster information could be incorporated into existing diabetes-care workflows without introducing excessive complexity, automation bias, or inappropriate reliance on algorithmic classifications (33, 34).

These considerations are directly relevant to the present study. The analysis included 95 participants, comprising 75 patients with diabetes and 20 healthy controls, and therefore represents a relatively small, single-center cohort. Although CGM provides dense longitudinal measurements for each participant and thus substantially more observations than the number of participants alone might suggest, repeated measurements from the same individual are not equivalent to independent subjects and do not eliminate limitations associated with cohort size. Accordingly, the identified clusters should not yet be assumed to represent stable phenotypes across the broader T1DM population. They were derived from the present cohort and have not undergone independent external validation; their reproducibility and generalizability to populations with different demographic and clinical characteristics, treatment strategies, or CGM devices therefore remain uncertain.

The current findings should consequently be regarded as a hypothesis-generating demonstration of the feasibility and potential value of CGM-based phenotyping rather than as evidence of a clinically deployable decision-support system. Future studies should validate the identified clustering structure in larger independent and multicenter cohorts, evaluate cluster stability and reproducibility, assess robustness to differences in patient characteristics and CGM data acquisition, and determine whether the identified phenotypes are associated with clinically meaningful outcomes. Subsequent prospective studies should then assess whether incorporating cluster information into clinical workflows provides measurable benefit without compromising clinician oversight. Only after such external validation and implementation studies could the potential role of CGM-derived phenotypes in clinical decision support be appropriately established.

Second, the stochastic nature of UMAP additionally means that, despite the use of a fixed random seed for reproducibility, results may be sensitive to parameter settings and should be interpreted with appropriate caution. Third, the clustering approach was based exclusively on CGM-derived glucose metrics, and did not incorporate potentially relevant clinical variables such as dietary patterns, physical activity, insulin regimen type, or comorbidity burden. Fourth, the cross-sectional design precludes assessment of phenotype stability over time or inference of causal relationships between cluster membership and clinical outcomes. Longitudinal studies are needed to assess the temporal stability of identified clusters and their associations with disease progression and treatment response. Validation studies in larger cohorts with diverse demographic and clinical characteristics are essential to evaluate the clinical utility of this phenotyping approach.

In conclusion, this exploratory study demonstrates that unsupervised machine learning applied to CGM data can identify six CGM-derived glycemic phenotypes within a cohort of individuals with T1DM. The identified exploratory glycemic clusters showed differences in glucose variability and hypoglycemia burden, together with preliminary evidence of differences in eGDR and triglyceride levels between selected T1DM and control group clusters. The control group served as an additional reference group. Liver enzyme differences did not reach statistical significance and should therefore be regarded as hypothesis-generating observations rather than established characteristics of the identified clusters and control group. These findings provide a proof-of-concept for CGM-based phenotypic stratification and support further investigation of data-driven approaches to characterizing glycemic heterogeneity in T1DM.

Importantly, the identified clusters should not be interpreted as validated biological or clinical T1DM subtypes. Their clinical relevance, reproducibility, and stability over time remain uncertain, particularly given the cross-sectional design, small cluster sizes, and absence of external validation. Future research should therefore evaluate these CGM-derived phenotypes in larger, independent, and demographically diverse cohorts, investigate their longitudinal stability, incorporate broader clinical and lifestyle variables, and determine whether cluster membership is associated with treatment response or clinically meaningful outcomes. Although such data-driven approaches may ultimately contribute to more individualized diabetes management, their potential to inform treatment strategies remains unproven and requires prospective validation. Accordingly, the approach described here should not be considered ready for direct clinical implementation without further rigorous validation.

## Data Availability

The data analyzed in this study is subject to the following licenses/restrictions: Data owner restricted public access. Requests to access these datasets should be directed to Dr. Jelizaveta Sokolovska, jelizaveta.sokolovska@lu.lv.
