# Supplementary material for: Distinct insulin sensitivity profiles revealed by clustering based on continuous glucose monitoring data in people living with type 1 diabetes
Source: Front Endocrinol (Lausanne). 2026 Sep 28;17:1963929. doi: 10.3389/fendo.2026.1963929 (PMC13654712; doi:10.3389/fendo.2026.1963929)
Supplement: Supplementary file 1 [file SupplementaryFile1.docx]

**Supplementary Materials**

**Figure S1. UMAP-derived CGM glycemic clusters.**

Numbers indicate the initial UMAP-derived clusters before consolidation into the final clinically meaningful phenotypic groups.

**Figure S2. Elbow plot showing K-means clustering distortion across candidate numbers of clusters (K)**

**Table S1. Clinical characteristics of patients in different glycaemic clusters and control subjects.**

|  | **Cluster “N”**  **N = 11** | **Cluster**  **“SeH”**  **N = 17** | **Cluster**  **“ModH”**  **N = 14** | **Cluster**  **“MiH”**  **N = 15** | **Cluster**  **“HAH”**  **N = 13** | **Cluster**  **“HAN”**  **N = 5** | **Control Cluster**  **“No”**  **N = 20** |
| --- | --- | --- | --- | --- | --- | --- | --- |
| **Female,**  **N (%)** | N=6  (54%) | N=8  (47%) | N=11  (79%) | N=10  (67%) | N=6  (46%) | N=3  (60%) | N=14  (70%) |
| **Age,**  **years** | 43.0 (33.0 - 55.0) | 47.0 (38.0 - 53.0) | 46.5 (35.0 -  54.5) | 37.0 (32.0 - 49.5) | 45.0 (35.0 - 49.0) | 35.0 (34.0 - 36.0) | 37.0 (34.25 - 41.0) |
| **Smoking,**  **N (%)** | N=5  (45%) | N=6  (35%) | N=5  (36%) | N=8  (53%) | N=9  (69%) | N=0  (0%) | N=5  (25%) |
| **Body mass index, kg/m^2^** | 25.50  (23.65 -  27.50) | 26.40 (23.70 - 28.40) | 25.80 (23.23 - 29.22) | 24.10 (23.25 - 26.80) | 23.80 (22.90 - 26.10) | 22.20 (21.70 - 23.70) | 23.65 (21.53 - 24.95) |
| **Length of diabetes, years** | 37.0 (23.0 - 41.0) | 22.0 (18.0 - 27.0) | 29.0 (23.3 -  32.8) | 25.0 (16.0 - 27.0) | 26.0 (13.0 - 33.0) | 11.0 (10.0 - 14.0) | 0 |
| **Hypertension,**  **N (%)** | N=7  (64%) | N=11  (65%) | N=9  (64%) | N=10  (67%) | N=7  (54%) | N=0  (0%) | N=3  (15%) |
| **Severe retinopathy,**  **N (%)** | N=7  (64%) | N=7  (41%) | N=8  (57%) | N=8  (53%) | N=8  (62%) | N=1  (20%) | N=0  (0%) |
| **CVD,**  **N (%)** | N=3  (27%) | N=5  (29%) | N=4  (29%) | N=1  (7%) | N=2  (15%) | N=0  (0%) | N=5  (27%) |
| **End-stage kidney disease,**  **N (%)** | N=2  (18%) | N=1  (6%) | N=1  (7%) | N=1  (7%) | N=2  (15%) | N=0  (0%) | N=0  (0%) |

| **HbA1c, %,** | 6.90 (6.20-7.45) | 9.80 (8.90- 11.20) | 9.10 (8.00- 9.38) | 8.10 (7.35- 9.60) | 7.90 (7.54- 8.50) | 6.10 (5.60- 6.50) | 5.15 (4.88- 5.33) |
| --- | --- | --- | --- | --- | --- | --- | --- |
| **eGFR**  **ml/min/1.73m2** | 96.17  (81.64-  107.86) | 93.83 (82.45- 103.30) | 72.79 (46.88- 111.85) | 98.02  (61.36-  109.22) | 99.66 (69.48- 107.87) | 111.28  (107.04-  117.99) | 104.46  (89.23-  109.77) |
| **Albuminuria,**  **mg/mmol** | 1.20 (0.90- 1.42) | 2.70 (0.63-  19.65) | 5.29 (0.93-  88.02) | 0.86 (0.54- 7.10) | 2.28 (0.79-  5.30) | 1.50 (0.90- 5.82) | 1.11 (0.48- 2.33) |
| **High-density**  **lipoprotein**  **cholesterol, mmol/l** | 1.84 (1.30- 2.09) | 1.79 (1.62-  2.36) | 1.79 (1.28-  2.12) | 1.74 (1.54- 2.01) | 1.67 (1.62-  2.09) | 2.34 (1.74- 2.74) | 1.71 (1.60- 2.04) |
| **Low-density**  **lipoprotein**  **cholesterol, mmol/l** | 2.45 (1.94- 2.88) | 2.83 (2.40-  3.57) | 2.45 (1.85-  3.08) | 2.80 (2.18- 3.33) | 2.50 (2.10-  3.40) | 2.69 (2.20- 3.36) | 2.89 (2.22- 3.34) |
| **Total cholesterol, mmol/l** | 4.38 (3.93- 5.16) | 5.07 (4.58-  6.39) | 4.80 (4.04-  5.46) | 4.94 (4.47- 5.46) | 4.92 (4.09-  5.49) | 5.30 (4.57- 5.47) | 5.21 (4.19- 5.40) |
| **Triglycerides,**  **mmol/l,** | 0.81 (0.74- 1.26) | 1.31 (0.82-  1.94) | 0.98 (0.76-  1.93) | 1.25 (0.91- 1.63) | 1.12 (0.84-  1.40) | 0.52 (0.50- 0.70) | 0.80 (0.67- 1.55) |
| **C-reactive protein, mg/l** | 0.69 (0.53 - 1.35) | 1.15 (0.69 -  3.17) | 2.00 (0.80 -  3.04) | 1.49 (0.61 - 2.01) | 0.83 (0.62 - 1.43) | 0.41 (0.40 - 1.09) | 0.44 (0.33 - 1.01) |

**arterial hypertension systolic blood pressure 140 mmHg (18.7 kPa) or higher, a diastolic blood pressure of 90 mmHg (12.0 kPa) or higher, or a history of antihypertensive drug use. Smoking status - currently smoking. CVD - history of acute myocardial infarction, coronary bypass or percutaneous transluminal coronary angioplasty, stroke, amputation, or peripheral vascular disease. Severe retinopathy - proliferative retinopathy or status post-laser photocoagulation of the retina.*

**Table S2.Characteristics of clusters based on glucose events.**

|  | **Cluster “N”**  **N = 11** | **Cluster**  **“SeH”**  **N = 17** | **Cluster**  **“ModH”**  **N = 14** | **Cluster**  **“MiH”**  **N = 15** | **Cluster**  **“HAH”**  **N = 13** | **Cluster**  **“HAN”**  **N = 5** | **Control**  **Cluster**  **“No”**  **N = 20** |
| --- | --- | --- | --- | --- | --- | --- | --- |
| **Average glucose mmol/L** | 7.4 ± 2.8 | 14.1 ± 5.9 | 11.0 ± 4.5 | 9.0 ± 3.4 | 8.8 ±4.2 | 6.4±2.7 | 5.7±0.9 |
| **Coefficient of variance %** | 37% | 39% | 40% | 38% | 49% | 43% | 16% |
| **Glucose management indicator %** | 6.6 | 9.3 | 8.0 | 7.2 | 7.1 | 6.1 | 5.7 |
| **% of time below target (Level 2 hypoglycemia)** | 1% | 1% | <1% | 1% | 3% | 3% | <1% |
| **% of time below target (Level 1 hypoglycemia)** | 8% | 2% | 2% | 4% | 10% | 19% | <1% |
| **% of time in target** | 75% | 23% | 42% | 60% | 51% | 69% | 98% |
| **% of time above**  **target (Level 1**  **hyperglycemia)** | 14% | 26% | 31% | 27% | 23% | 7% | 1% |
| **% of time above**  **target (Level 2**  **hyperglycemia)** | 2% | 48% | 24% | 9% | 13% | 2% | <1% |

| **All low glucose events** | 24 | 11 | 45 | 15 | 70 | 37 |  |
| --- | --- | --- | --- | --- | --- | --- | --- |
| **Average low glucose events per patient** | 2 | 1 | 3 | 1 | 5 | 7 | - |
| **All low glucose event duration, minutes** | 2190 | 2055 | 1020 | 1365 | 6765 | 2475 | - |
| **Average low glucose events duration,**  **minutes per patient** | 199 | 121 | 73 | 91 | 520 | 495 | - |
| **Average low glucose events duration, minutes per event** | 91 | 187 | 23 | 91 | 97 | 67 | - |
